# Sources of Dietary Sodium in Food and Beverages Consumed by Spanish Schoolchildren between 7 and 11 Years Old by the Degree of Processing and the Nutritional Profile

**DOI:** 10.3390/nu10121880

**Published:** 2018-12-03

**Authors:** Esther Cuadrado-Soto, África Peral-Suarez, Aránzazu Aparicio, Jose M. Perea, Rosa M. Ortega, Ana M. López-Sobaler

**Affiliations:** 1 Department of Nutrition and Food Science, Faculty of Pharmacy, Complutense University of Madrid, Plaza Ramón y Cajal S/N, 28040 Madrid, Spain; africper@ucm.es (Á.P.-S.); araparic@ucm.es (A.A.); rortega@ucm.es (R.M.O.); asobaler@ucm.es (A.M.L.-S.); 2 UCM Research Group: VALORNUT-920030, 28040 Madrid, Spain; jmpereas@ucm.es; 3 Faculty of Health Sciences, Alfonso X El Sabio University, Avda. Universidad, 1, Villanueva de la Cañada, 28691 Madrid, Spain

**Keywords:** sodium, food processing, ultra-processed, Spanish children, food environment, discretionary food, salt

## Abstract

Excessive salt intake has negative effects on health and persists as a dietary problem in Spanish children. However, the analysis of dietary sodium sources has not been extensively studied. A group of 321 children between 7 and 11 years old from five Spanish regional communities was studied. A three-day dietary record was used to determine the contribution of food and beverages to dietary sodium intake. The food consumed was classified based on the level of processing (NOVA classification) and the nutritional profile. Boys consumed more dietary sodium and sodium from ultra-processed food (UPF) than girls (*p* < 0.05). The main sources of dietary sodium from discretionary food were meat and meat products (25.1%), some ready-to-eat and pre-cooked dishes (7.4%) and sugars and sweets (6.3%). More than 4/5 of the total dietary sodium consumed came from processed foods (PF) and UPF. Ready-to-eat and pre-cooked dishes (14.4%), meat and meat products (10.6%), and cereals (10.2%) were the most relevant UPF. These results demonstrate that a key point for Spanish children is a reduction in the sodium content in PF and UPF, whether these foods are for basic or discretionary consumption. Furthermore, a decrease in the frequency and the quantity of discretionary food consumption should be encouraged.

## 1. Introduction

Currently, excess sodium in the diet is a common problem worldwide [1]. High intake of dietary sodium is associated with an increased risk of hypertension, cardiovascular disease and stroke, entailing great health expenditures [2,3,4].

In this sense, in 2005, experts of the European Food Safety Authority (EFSA) Working Panel concluded that, although it was not possible to determine a tolerable level of sodium intake for the population, the excess intake of dietary sodium is associated with cardiovascular damage and a detrimental effect on health [5].

On the other hand, in 2003, the World Health Organization (WHO) previously established the reference of a maximum intake of 5 g/day of salt in adults as a global target [6]. In 2012, the WHO extended this recommendation to children, whose sodium reference value is proportional to the energy they consume in relation to adults [1]. However, different studies carried out in European countries showed that the majority of the school population exceeds the recommended amount of salt [7]. For example, the salt intake was 7.4 g/day and 6.7 g/day in boys and girls aged 6–18 years in Italy, respectively [8], and 5.7 g/day in children aged between 7 and 10 in France [9].

The lack of compliance with the salt recommendation in the population could be related to the fact that people are not conscious of the amount of salt they consume, and efforts to reduce their intake are limited by the sodium content of processed products [10]. Furthermore, the problem in children is even higher, since exposure to these foods can be maintained as adults because different habits acquired by children at an early age follow a pattern of maintenance throughout their lives [11]. Additionally, it has been observed that preference for salty foods in childhood comes from learned experiences in addition to biological factors [12,13]. To improve this situation it is necessary to identify the sources of sodium intake and to develop policies and public strategies to reduce the of salt intake in the population, especially in children, which will have great advantages in terms of public health as a cost-effective strategy [4]. In this sense, in Spain, to reduce hidden salt in food, the Spanish Agency for Consumer Affairs, Food Safety and Nutrition (AECOSAN) have recently established agreements with the industry in order to modify the salt content of basic foods in the diet such as bread, and discretionary foods that are processed (PF) and ultra-processed (UPF), such as processed meat, snacks, and canned soups [14].

In literature, foods have been usually categorized according to their special nutritive contribution, grouped into fruits, vegetables, cereals, etc. However, in the case of sodium, it could be more useful to identify their sources according to the degree of processing. Identifying the quantity of sodium that comes from foods with a higher industrial processing could be useful to distinguish the sodium that can be modified through food reformulation either by decreasing the amount of sodium in foods or by reformulating the salt (sodium chloride) to other types of salts as those that incorporate iodine or iron, which intakes are deficient in children [15,16]. Another way to classify foods is to consider if the foods are habitual or discretional in the diet because it allows us to distinguish if the sodium comes from foods and beverages whose consumption should not be decreased, because they are basic foods, or if it comes from foods whose consumption should be exceptional and is not being so [17].

Having in mind all the above and that there are no data available in Spain regarding the sources of dietary sodium consumed by schoolchildren that take into account food and beverage processing or nutritional profiles, the aim of this work is to determine the main sources of sodium intake consumed by the Spanish child population between 7 and 11 years old, considering the type of food and beverages based on the degree of processing and the nutritional profile.

## 2. Materials and Methods 

The design of the study has been previously described [18]. This trial was registered at clinicaltrials.gov as NCT03465657.

The study was conducted in accordance with the guidelines laid down in the Helsinki Declaration. Written consents were obtained and signed by the children’s parents or legal guardians. The final protocol (12/394-E) of the study was approved by the Ethics Committee of the San Carlos Clinic Hospital, Madrid (Spain). The manuscript does not contain clinical studies or patient data.

### 2.1. Study Design

A cross-sectional study was conducted between February 2014 and November 2017. Sixteen schools were contacted at random, of which eight schools from five Spanish provinces participated. Four of the schools were in the respective capitals of each participating province, and the rest were in semiurban cities (<50,000 inhabitants). 

### 2.2. Subjects

**Figure 1:**
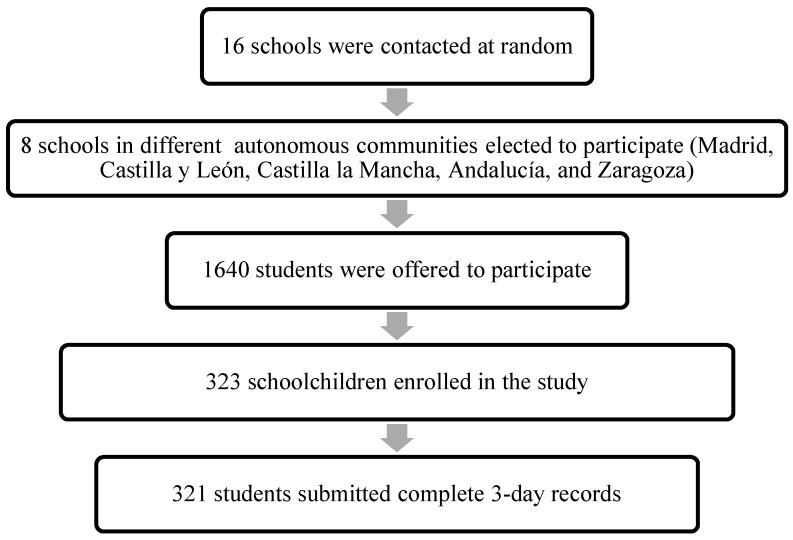
Flowchart of recruitment and selection of the participants.

One investigator contacted the schools. Once the school’s participation was confirmed by the director, an appointment was arranged with parents who were interested in the study. They attended an informative talk in which they provided signed consent for their son or daughter to participate in the research project. This meeting clarified all possible questions from the parents and informed them about the study in detail. All participants entered as volunteers, and the children provided complete information about their health data and a dietary questionnaire. Subsequently, a visit was made to the school in which the students returned the questionnaires, and some anthropometric measurements were made. Of the 1640 children who were offered to participate in the study, 323 were finally included (Figure 1).

The exclusion criteria were: not being present on the day of the visit to the school, having a disease that could affect the results, having altered eating habits and having incomplete questionnaires. Of the 323 participants who enrolled in the study, two of them did not complete the dietary questionnaire, resulting in a final sample of 321 schoolchildren.

### 2.3. Collection of Dietary Data

A three-day dietary record (two weekdays and one weekend day) was used to determine all foods and beverages consumed by children during that time period. 

The questionnaires were completed by the parent or guardian of the schoolchildren, who were required to record all the food, beverages and supplements consumed during the pre-established period. For the proper completion of the registration, parents or guardians were informed in a clear and precise way about how they should record all the information in detail, including the methods of food preparation and the ingredients in dishes and recipes when possible, and the brands of commercial products.

The format used in the register was structured by days and different meals (breakfast, lunch, snack, dinner, and “between meals”); the time, place and menu were also noted. The importance of mentioning the food consumed between meals (snacks, sweets, etc.), as well as the consumption of bread, or ingredients used to prepare dishes, sweeteners, etc. was stressed. From this register, the observed food intake of the population was obtained.

In addition, to minimize mistakes after data collection, all interviews were reviewed by the study dietitians to assess unrealistic portion sizes and fluid intake, inadequate details, and typing errors.

Sodium and energy intake from the food and beverages consumed were calculated using the DIAL software version 3.0.0.12 (Alce Ingeniería, Madrid, Spain) [19]. It allowed us to determine the individual intake of food, energy and nutrients, through the data from the Spanish Food Composition Tables (FCT) [20]. These FCT were supplemented with additional nutrient composition data for specific brands (for example normal or low-sodium salt content) and fortified foods. Only food and beverage intake were analyzed without considering dietary supplements or table salt or salt used in cooking.

### 2.4. Sodium Sources and Food Grouping

An analysis of the total amount of sodium provided by all the foods eaten by the population was performed. The sodium content specified in each food and beverage was added according to its intake by everyone in the group. This sodium was compared to the total of sodium consumed, which was obtained from the sum of the total sodium ingested by all participants during the three days. The percentage of sodium from each group was calculated as follows: (sum of sodium from food group (mg)/total sum of sodium from all foods (mg)) × 100 [21].

Food groups and subgroups that contributed the most to sodium intake were identified. The sources of sodium were also analyzed according to the origin, and foods that were discretionary foods or core foods in the diet were analyzed based on the level of processing. To establish sodium sources by food groups, foods collected in the dietary surveys were classified into 14 food groups and 64 subgroups.

### 2.5. Food Classification Based on the Nutritional Profile

To classify foods according to their nutritional profile we use the core/discretionary classification which has been widely used [22,23,24,25]. Because in Spain there are no recommendations to classify foods according to their nutritional profile, we used the following principles established in the Australian Food Guidelines 2011–2013 and its supporting documents were used [26]. We classified foods and beverages into two groups, core and discretionary. By dividing foods into these two categories, we could assess whether dietary sodium comes from foods for daily or optional consumption.

This classification is based on the consideration of 5 basic food groups (core foods): (1) cereals, (2) vegetables and pulses, (3) fruits, (4) milk, yogurts and cheeses or alternatives, (5) lean meats and chicken, fish, eggs, tofu, nuts, seeds and other pulses. For questionable items, a review of the detailed list of discretionary foods provided by the Australian Bureau of Statistics was conducted [27].

On the other hand, discretionary foods are distinguished by their high content of saturated fats, added sugars, salt and/or alcohol. Therefore, they are described as dense in energy, and their consumption must be occasional and in small quantities.

In most cases, foods were classified according to this methodology at the group level and not at the food level. For example, all soft drinks were classified as discretionary, including sweetened or light drinks. In other food groups, depending on the foods included, items were reviewed one by one. For example, fruit juices were classified as core foods, but other juice drinks were classified as discretionary foods.

To facilitate classification, cut-off points were established for the fat and sugar content of certain types of foods, taking into account the value of these nutrients in discretionary foods (cookies, ice cream, sweetened drinks, chips, etc.). For example, cakes are considered discretionary foods and have average sugar and lipid content of 20 g and 15 g per 100 g, respectively, so the cut-off point was set at 15 and 20% for total fats and sugars, respectively. All those foods classified in the group of cakes such as buns, muffins, etc. used this cut-off point. This facilitated the classification of foods into groups with greater food diversity.

The principles used for the classification of foods included in some heterogeneous food groups that were based on the nutritional profiles of the foods. These principles included the following:

- Food fortification did not alter whether food was classified as basic or discretionary. For example, sugar-sweetened beverages with added vitamins were considered discretionary.
- For breakfast cereals, discretionary foods were defined as breakfast cereals with >30 g of simple sugars per 100 g.
- For breakfast cereals with added fruit, discretionary foods were defined as breakfast cereals with >35 g of simple sugars per 100 g.
- For dishes derived from cereals (e.g., prepared sandwiches) and some ready-to-eat meals (burgers, wrappers, sushi, pizzas, kebab, sausages), discretionary foods were defined as those that exceeded 5 g of saturated fat per 100 g. The subgroup of prepared and pre-cooked dishes was a very varied group in which food had to be classified by the product and not by the group.
- Salt biscuits were classified as basic if they contained less than 430 kcal/100 g.
- Canned soups were considered discretionary because of the high sodium content per 100 g.

The total amount of beverages and foods consumed by each participant was determined based on whether the foods were core or discretionary foods.

### 2.6. Food Classification Based on the Degree of Processing 

We used the NOVA classification. The NOVA system organizes food according to the degree of processing. Products were grouped into four categories: “minimally processed, processed culinary ingredients, PF and UPF.” This food classification process has been extensively explained in the literature and is used even by institutions, such as the PAHO (Table 1) [22,28,29].

### 2.7. Sociodemographic and Anthropometric Data

The participants completed a general questionnaire with sociodemographic and health data. The questionnaire included questions such as the date of birth, parents’ academic level, and health status (e.g., use of medication and presence of chronic or acute diseases, special diets). 

Weight and height were determined with a digital balance (range 0.1–150 kg; accuracy 100 g; Alpha; Seca, Igni, France) and a digital stadiometer (70–205 cm; 1 mm; Harpenden Pfifter, Carlstadt, NJ, USA). Anthropometrists measured children, who were barefoot and in light clothing or underwear. The body mass index (BMI) was then calculated. All measurements were determined in duplicate.

### 2.8. Validity of the Dietary Assessment Method

To assess the validity of the reported energy intake (EI) at the individual level, the EI:BMR ratio was calculated. To calculate the basal metabolic rate (BMR), we used the Schofield equations [30], which take into account age, sex, body height and weight. This ratio was compared with the appropriate cut-off point according to the Goldberg cutting method [31]. The plausibility of the intake was determined following the steps proposed by EFSA [32]. We used specific reference values for coefficients of variation in terms of energy intake (EI), basal metabolic rate and physical activity, as indicated by Black [33]. The rate of under- and over-reporters was calculated with the EI:BMR ratio. We identify misreporters (under- and over-reporters) by the ratio obtained in comparison to the previously established cut-off points. We used the group levels to calculate the overall bias of the reported energy intake. Under-reporters were identified as those with EI/BMR ratios up to 1.06–1.07, while over-reporters were identified by EI/BMR ratios above 2.26–2.11, depending on the subject’s age and sex. In our analysis of sodium sources, we present data from the total sample. 

### 2.9. Statistical Processing of Data

We tested categorical variables using the *χ*^2^ test to assess differences between the sample characteristics. We used Student’s *t*-test for continuous and parametric variables and the Mann-Whitney *U* test for nonparametric variables. The Kolmogorov-Smirnoff one-sample test was used to check whether the dietary sodium intake followed a normal distribution and to decide between parametric and nonparametric analysis. Dietary sodium intake data were presented in the total population and stratified by sex, using the mean and standard deviation and the median and interquartile range. Gender differences were measured using Student’s *t*-test or the Mann-Whitney test. The percentage of sodium in different food groups was also analyzed. The contribution of data for energy and dietary sodium intake of foods according to different classifications were expressed as a percentage of total energy and sodium intake. The results for all variables were expressed as mean ± standard deviation (SD), medians with interquartile range or percentages where appropriate. The significance level was set at *p* < 0.05. The analysis was conducted using the statistical software SPSS version 22 (SPSS, Inc., Chicago, IL, USA). 

## 3. Results

### 3.1. Descriptive

Of the 323 children (8.8 ± 1.2 years) who agreed to participate in the study, 321 schoolchildren completed the dietary data (156 girls). Table 2 shows personal, demographic and socioeconomic distribution of the participants, along with their anthropometric measurements in the total sample and by sex. We did not find significant differences in sociodemographic variables according to sex. Overall, 2.2% of the schoolchildren were under-reporters, and 7.5% overestimated their diet.

### 3.2. Dietary Sodium and Energy Intake by Sex through Two Food Classification Systems

Sodium and energy intake by sex and by selected food categories are presented in Table 3. It was observed that energy, total dietary sodium and sodium form UPF intake was significantly higher in boys in comparison to girls. On the other hand, no differences according to the sex were found in sodium intake in the rest of the food groups established (*p* > 0.05). 

### 3.3. Top Sources of Dietary Sodium

Table 3 also shows how core foods contributed to slightly more than half of the total sodium intake with respect to discretionary foods. In addition, it was observed that UPF foods and PF contributed more than 80% to the total dietary sodium intake, followed by minimally processed foods and processed culinary ingredients. 

#### 3.3.1. Sources of Dietary Sodium According to Food Groups and Nutritional Profile

**Figure 2:**
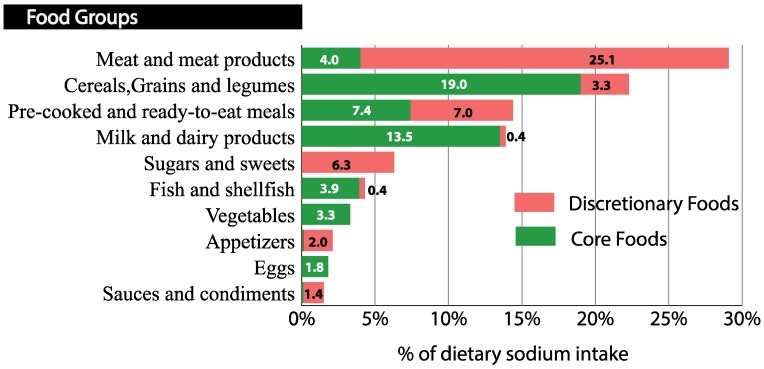
Sources of dietary sodium based on their classification as core or discretionary foods (*n* = 321). Food groups and dietary sodium intake were derived from a 3-day food record. Foods were classified based on their nutritional profile as core foods or discretionary foods following the Australian Dietary Guidelines, 2013 [26]. The sodium contribution was expressed as a percentage of the total dietary sodium intake.

In terms of food groups and nutritional profile, meat and processed meat group contributed to the highest proportion of dietary sodium intake (29.1%) follow by cereals group and pre-cooked and ready-to-eat meals. The fourth group that contributed the most to sodium intake was milk and dairy products, and sugars and confectionery were the fifth largest contributor to higher dietary sodium intake. These five groups represented 86.1% of the total dietary sodium intake in the population (Figure 2).

Additionally, discretionary foods in meat and meat products, sugars and sweets, appetizers and sauces and condiments contributed 85–100% to the total sodium intake of these groups, while core foods provided the most dietary sodium in cereals and milk and dairy products (85.2–97.1%) (Figure 2).

#### 3.3.2. Sources of Dietary Sodium According to Food Groups and Degree of Processing

**Figure 3:**
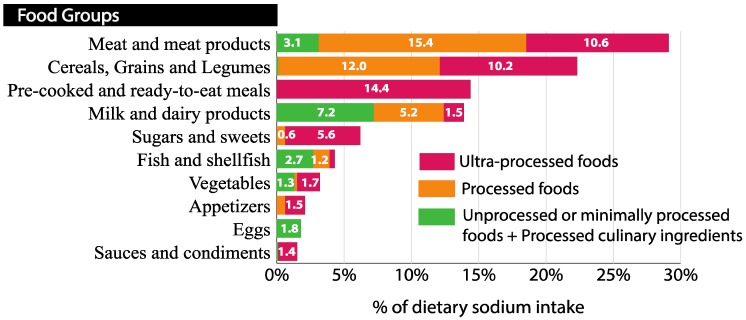
Sources of dietary sodium based on the degree of processing of food (NOVA classification) (*n* = 321) [22,28,29]. Contribution of the sodium content in different food groups to the total sodium dietary intake. Food groups and dietary sodium intake were derived from a 3-day food record. Foods were classified based on the degree of processing according to the NOVA system. They were organized into three groups in this figure: unprocessed or minimally processed foods + processed culinary ingredients, processed foods and ultra-processed foods. The sodium contribution was expressed as a percentage of the total dietary sodium intake.

Considering food groups and the degree of processing, UPF provided 47.8% of the dietary sodium consumed and with PF provided more than 4/5 of the dietary sodium intake. According to the degree of processing, PF contributed to a greater percentage of sodium intake than UPFs or unprocessed or minimally processed foods (NP + MPF) and PCI in meat and meat products and cereals group (53%). Meanwhile, the sodium intake that came from UPF contributed in a higher way than the other NOVA groups in ready-to-eat dishes, sugars and pastries, vegetables, appetizers and sauces and condiments groups (56.5–100%) (Figure 3). 

#### 3.3.3. Sources of Sodium Intake According to Food Subgroups, Nutritional Profile, and Degree of Processing

Table 4 shows the subgroups of foods ordered based on their contribution to the total dietary sodium intake and classified based on their basic or discretionary consumption in the diet and the NOVA classification. It was found that white bread, Serrano ham, cured sausages and milk were the food subgroups that contributed more to sodium intake independently of the classification considered.

## 4. Discussion

To the best of our knowledge, the present study identified the main contributors to dietary sodium intake, excluding sodium from table salt, in Spanish children between 7 and 11 years old considering the degree of processing and the nutritional profile. PF and UPF contributed almost all dietary sodium. The average dietary sodium intake in Spanish schoolchildren aged between 7 and 11 years old was 2026 mg/day (5.1 g salt/day). In a previous analysis of a subsample of our study population, Aparicio et al. [18] obtained from a 24-h urine sample from each of the participants. They found that 84.5% of subjects under 10 years of age consumed >4 g salt/day, and 66.7% of those aged >10 years of age consumed >5 g salt/day. The average amount of salt intake of the study population was 8.3 g/day and 7.2 g/day in boys and girls, respectively. The differences found between both studies could be explained by the fact that we did not consider table salt or cooking salt because of the difficulty and error in reporting the exact amount of salt used [34]. Additionally, this fact could justify the differences found between our results and the results of the ENALIA study [35], where table salt and salt added during cooking were considered. In this study, the average intake of sodium in children aged 9 to 13 years was 2519 ± 577.5 mg/day and 2177 ± 571.7 mg/day in boys and girls, respectively. In our study, we included misreporters, similar to the ENALIA study. The best way to address under-reporters in analyses is not entirely clear [36,37]. Some studies exclude under-reporters from the dataset, but this approach not only reduces statistical power, but also introduces selection biases.

In the present study, boys had higher total energy, total dietary sodium, and sodium intake from UPF than girls, although no differences in the energy intake from UPF were observed. This means that boys chose UPF foods with higher sodium density. Our results are similar to those observed by Aparicio et al. [18] who found a higher 24-h urinary sodium excretion in boys than in girls, while the ENALIA study found a higher prevalence of excessive sodium intake in boys [35]. One explanation for this could be that boys prefer more palatable foods. For example, in a study by Desor et al. [38], boys had a greater preference for sweet tastes than girls, although there was no difference in their preference for salty tastes. Further investigation should be performed in this sense. 

With reference to sources of sodium by nutritional profile (core/discretionary), we found that in the groups of the cereals and dairy products the core foods provided the most dietary sodium. It is recognized that some of the foods classified as core foods contain a high amount of sodium, such as bread and dairy products [39]. However, it is not advisable to reduce the intake of these foods because they promote health benefits because they are dense in other nutrients. It was notable to find pre-cooked foods among the core foods. In our study, we included some prepared and pre-cooked pizzas and dishes that could be considered basic in the diet because of their low saturated fat content. These foods were classified within a food group with a very heterogeneous composition, and the overall food group was not considered discretionary. These foods were classified according to their saturated fat content regardless of their sodium content. Perhaps sodium should also be considered in these foods, and a cut-off point should be set for this risk nutrient to classify pre-cooked food as core or discretionary. In core foods, we consider it essential for the industry to reformulate the sodium content. It is also important to educate the population not to avoid these foods, but to encourage them to look at the labels and select those options that are lower in this mineral [26]. 

Regarding discretionary foods and drinks, the energy provided attracted attention because it accounted for almost one-third of the energy consumed by children (31.3%). These foods were denser in sodium than basic foods, accounting for about half of the dietary sodium. The major contribution of sodium from discretionary foods and drinks came from Serrano ham and processed meats. Unlike in other countries, processed meat in Spain is the main source of sodium in children, compared to cereals in the United Kingdom, Finland, Colombia, and Australia [40,41,42,43]. It would be advisable to reduce the sodium content in discretionary foods, as well as to reduce the intake of these foods to more occasional consumption and in smaller portions. These foods should only be consumed in addition to core foods, depending on age, gender, sex, height, physical activity level, and the presence of excess weight [44].

On the other hand, considering the degree of processing, UPF provided almost half of the dietary sodium consumed (47.8%). UPF in conjunction with PF provided more than 4/5 of the dietary sodium intake. In the review by Sarmugam and Worsley [45], in seven of nine studies that asked about PF as a source of salt in the diet, more than 70% of respondents recognized them as important sources of salt. Since PF are easily identifiable by the population, they may be a good starting point for addressing recommendations. In the latest update of the Dietary Guidelines for the Spanish population by the SENC [46] the need to moderate the population’s salt intake to less than 6 g/day was noted. However, they did not mention the contribution of UPF to dietary sodium intake, and this should be reported. 

By food subgroups, the main sources of sodium obtained were white bread (11.6%), Serrano ham (8.2%), cured cold meats (6.6%) and milk (6.5%). Our results are consistent with those obtained in other Mediterranean countries, such as Greece and Portugal. In the GRECO study [47], the greater sources of sodium in children’s diets were pizza, white cheese, processed cereals, yellow cheese, and bread. In a sample of Portuguese adolescents [48], the major food sources of sodium were cereal and cereal products, meat products and cheese. Fast food contributed to 9% of the total sodium intake, and all the major food sources were PF or UPF, except for milk. Recognizing whether a food has only natural sodium (as in the case of milk or unprocessed foods) or also has sodium from the addition of salt to the food (as in the case of cheeses or PF/UPF) can serve to indicate to the population in which foods it is especially important to look at the sodium content on the label when buying. For example, in the case of milk and dairy products, the sodium content will be similar in different kinds of milk. However, the sodium content of cheeses can vary widely, and it is essential to look at their content on the label.

Our study has some limitations. First, the sample is not a representative sample of the Spanish child population. However, this is a broad sample of convenience that includes many children from diverse socioeconomic status, representing the rural and urban environments and different Spanish autonomous communities. Anyway, if we want to generalize our findings to the whole Spanish population, we should do it with caution.

Other limitations are inherent in the use of dietary surveys to estimate intake, and there is a possibility that the FCT may not accurately record the sodium content of foods, especially PF and UPF. On the other hand, the FCT needs to be updated following agreements with the food reformulation industry to accurately reflect the content of sodium and other nutrients. In this respect, the Spanish FCT used [20] were very up to date and additional composition data for specific brands were taken into account.

Among the strengths of our work is the use of a three-day dietary record. Although this questionnaire is not a good method for calculating total sodium intake (the gold standard is the 24-h urine collection [5]), it is used as an adequate direct assessment for estimating the dietary sources of sodium as indicated by WHO [49]. Dietary data from this questionnaire were detailed by parents and children and allowed the description of the sources of sodium in foods and beverages, which is difficult in this age group.

We also used two novel food classification systems that defined the degree of processing and the nutritional profile of foods based on whether their consumption was basic or discretionary in the diet. Both are useful classifications, given that not all PF and UPF products are discretionary and not all core foods are unprocessed. Also, we identified misreporters, as these could be used in the sensitivity analysis of the data [36,37].

Our data confirm the need for a reformulation of the sodium content of foods that affect children’s total dietary sodium intake. Foods in which it is easier to achieve a reduction in sodium content at an industrial level are foods that incorporate salt in their processing (PF or UPF foods). However, in countries such as Spain, the reformulation of sodium content by food group may not be sufficient, and consumers need to be aware of the need to reduce salt intake on their own [50]. Therefore, the best option for children to reduce their sodium intake below the recommended maximum levels is to combine the reformulation of PF and UPF foods to have lower sodium levels, with nutritional education in this area and to change the food environment. 

## 5. Conclusions

The results of this study demonstrate that PF and UPF provide significantly more dietary sodium than less PF in Spanish children, and they dominate the contribution to dietary sodium intake. Also, it was found that discretionary foods and beverages provide a large proportion of dietary sodium (and energy), contributing with almost half of the dietary sodium intake. Therefore, in order to achieve a reduction of sodium intake, it is suggested that the industry reformulate the sodium content of PF and UPF, either they are core and discretionary foods. Furthermore, regarding core foods, the population is encouraged to read the nutrition labeling properly and to choose those brands with lower sodium content while still consuming this type of food. On the other hand, there is a need to raise awareness among schoolchildren, particularly boys, and their parents about the large contribution of discretionary foods and UPF to sodium and energy intake and about the benefits of decreasing the intake of these foods to more occasional consumption.

## Figures and Tables

**Table 1:** Summary of the NOVA classification [22,28,29].

Nova Group	Characteristics	Processes Included
Group 1: Unprocessed or minimally processed foods (NP + MPF)	Minimally processed foods and beverages, without adding new ingredients from other groups.	To conserve food and make it suitable for storage (freezing, packaging, fractioning…), facilitate its culinary preparation (crushing, filtering, roasting…) and improve its nutritional quality.
Group 2: Processed culinary Ingredients (PCI)	These ingredients are extracted from group 1 foods or nature. They are usually not consumed on their own. *	Pressing, refining, grinding, milling, and spray drying.
		May contain additives such as preservatives or stabilizing agents.
Group 3: Processed foods (PF)	They are relatively simple products produced by adding sugar, oil, salt or other substances from Group 2 to Group 1 foods.	Addition of substances like sugar, oil or salt to group 1 foods.
		Non-alcoholic fermentation (breads and cheese).
		May contain additives such as preservatives or stabilizing agents.
Group 4: Ultra-processed food and drink products (UPF)	These are industrially manufactured foods and beverages that usually contain five or more substances as ingredients. They contain some of the ingredients present in processed foods (salt, sugar, oils and fats); however, UPF also includes ingredients that are not commonly used in cooking preparations, such as hydrolyzed proteins, modified starches and hydrogenated oils and additives.	Several industrial processes are used without equivalents in domestic or traditional cuisine.
		The main objective of industrial ultra-processing is to create ready-to-eat, ready-to-drink or ready-to-heat products that can replace unprocessed or minimally processed foods and freshly prepared dishes.

* For this study, discretionary salt has been excluded from studying dietary sodium exclusively.

**Table 2:** Personal, demographic and socioeconomic data of a random sample of Spanish schoolchildren between 7 and 11 years old.

Variables	Categories	Total % (*n*) *	Girls % (*n*) *	Boys % (*n*) *	*p* **
*n*		100 (321)	51.4 (165)	48.6 (156)	
Age (years)		8.8 ± 1.2	8.9 ± 1.2	8.8 ± 1.2	0.686 ***
Age groups					
	7–8 years	42.4 (136)	43.0 (71)	41.7 (65)	0.805
	9–11 years	57.6 (185)	57.0 (94)	58.3 (91)	
Residence					
	>50,000 inhabitants				
	Capital of the province	43.3 (139)	44.2 (73)	42.3 (66)	0.727
	<50,000 inhabitants	56.7 (182)	55.8 (92)	57.7 (90)	
Annual household income					
	Less than 18,000 €	27.0 (86)	29.9 (49)	23.9 (37)	0.568
	18,001–36,000 €	17.9 (57)	19.5 (32)	16.1 (25)	
	36,001–48,000 €	21.3 (68)	19.5 (32)	23.2 (36)	
	48,000 €	19.7 (63)	17.7 (29)	21.9 (34)	
	DK/NA	14.1 (45)	13.4 (22)	14.8 (23)	
Father’s education level					
	No school/primary school	24.0 (75)	22.2 (36)	26.0 (39)	0.190
	Secondary/VT	40.1 (125)	46.3 (75)	33.3 (50)	
	Graduated	31.1 (97)	26.5 (43)	36 (54)	
	Master’s/Ph.D.	4.8 (15)	4.9 (8)	4.7 (7)	
Mother’s education level					
	No school/primary school	18.2 (58)	17.8 (29)	18.7 (29)	0.448
	Secondary/VT	38.4 (122)	41.1 (67)	35.5 (55)	
	Graduated	38.7 (123)	38 (62)	39.4 (61)	
	Master’s/Ph.D.	4.7 (15)	3.1 (5)	6.5 (10)	
Anthropometric measurements					
Weight (kg)		35.4 ± 8.5	35.0 ± 8.5	35.7 ± 8.6	0.55 ***
Height (cm)		136.9 ± 9	136.2 ± 9.6	137.7 ± 8.4	0.144
BMI		18.7 ± 3.2	18.7 ± 3.1	18.7 ± 3.3	0.761 ***

BMI: Body Mass Index. DK/NA: Don’t Know/No Answer. VT: Vocational Training. * Values are presented as mean ± standard deviation (SD) or as a percentage with the number of subjects (n), ** tests of significance between groups were based on the chi-squared test, *** tests of significance between gender groups were performed with the Mann-Whitney test for independent samples.

**Table 3:** Distribution of dietary sodium (mg/day) and energy intake (kJ/day) and contribution to sodium and total energy intake (%) by classifying foods based on the level of processing (NOVA system) or the nutritional profile as core or discretionary foods.

Dietary Component	Food Classification	Total Population (*n* = 321)			Girls (*n* = 165)			Boys (*n* = 156)			
		Mean ± SD	P50 (P25,P75)	% total	Mean ± SD	P50 (P25,P75)	% total	Mean ± SD	P50 (P25,P75)	% total	*p*
Energy	Total	8761 ± 1347	8778 (7853,9791)	100	8565 ± 1372	8485 (7560,9678)	100	8950 ± 1301	9083 (8092,9904)	100	0.011
(kJ/day)	CF *	6021 ± 1121	6033 (5096,6740)	68.7	5904 ± 1119	5949 (5042,6550)	68.9	6130 ± 1118	6175 (5196,6899)	68.5	0.745
	DF	2745 ± 954	2686 (2013,3431)	31.3	2660 ± 962	2607 (1915,3351)	31.1	2821 ± 939	2751 (2074,3467)	31.5	0.157
	NP + MPF	3163 ± 828	3105 (2598,3711)	36.1	3117 ± 824	3059 (2548,3694)	36.4	3209 ± 833	3159 (2632,3745)	35.9	0.890
	PCI *	1042 ± 427	975 (749,1301)	11.8	1038 ± 439	975 (699,1310)	11.9	1050 ± 411	975 (774,1289)	11.7	0.597
	PF *	1452 ± 623	1423 (975,1787)	16.3	1418 ± 636	1397 (929,1741)	16.3	1485 ± 611	1452 (1050,1870)	16.3	0.296
	UPF	3138 ± 1155	3079 (2377,3870)	35.8	3033 ± 1121	3046 (2218,3665)	35.4	3238 ± 1180	3209 (2423,3962)	36.1	0.257
Dietary sodium	Total *	2026 ± 504	1990 (1676,2309)	100	1952 ± 461	1926 (1656,2217)	100	2099 ± 530	2044 (1743,2391)	100	0.010
(mg/day)	CF *	1090 ± 306	1057 (873,1271)	53.8	1059 ± 301	1025 (852,1237)	54.2	1120 ± 309	1080 (910,1292)	53.4	0.063
	DF *	937 ± 382	865 (637,1174)	46.2	893 ± 350	847 (620,1118)	45.8	978 ± 406	903 (661,1201)	46.6	0.110
	NP + MPF	337 ± 114	337 (261,411)	16.6	335 ± 117	337 (252,414)	17.2	340 ± 113	335 (277,403)	16	0.720
	PCI *	1.12 ± 2.1	0.02 (0.1,33)	0.1	0.87 ± 1.55	0.01 (0.1,1)	0.1	1.36 ± 2.5	0.02 (0.1,6)	0.1	0.188
	PF *	730 ± 380	680 (448,944)	35.5	712 ± 360	706 (423,942)	35.9	747 ± 398	643 (462,946)	35	0.688
	UPF *	970 ± 394	921 (679,1216)	47.8	916 ± 378	881 (673,1123)	46.9	1021 ± 403	999 (687,1260)	49	0.017

CF: Core food, DF: Discretional food, NP + MPF: Unprocessed or minimally processed foods, PCI: Processed culinary ingredients, PF: Processed foods, UPF: Ultra-processed foods. SD: Standard Deviation. * Does not follow a normal distribution.

**Table 4:** Contribution to energy and dietary sodium intake (%) based on the food subgroups by their nutritional profile and their processing level.

Classification of Foods According to Their Nutritional Profile				Classification of Foods According to Their Processing Level			
Sodium Ranking		% Energy	% Sodium	Sodium Ranking		% Energy	% Sodium
Core Foods and Beverages				Unprocessed or Minimally Processed Foods and Beverages			
1	White Bread	6.9	11.6				
2	Milk	7.7	6.5	1	Milk	7.7	6.5
3	Pizzas	2.0	4.8	2	Eggs	2.0	1.8
4	Sliced Bread	1.9	3.6	3	Poultry meat	2.6	1.2
5	Pre-cooked and ready-to-eat meals	1.1	2.6	4	Fresh shellfish and mollusks	0.5	1.2
6	Semicured and cured cheeses	1.5	2.6	5	Bovine meat	2.4	1.0
7	Yogurts and fermented milk	3.0	2.1	6	Whitefish	0.6	0.9
8	Vegetable preserves	0.3	1.9	7	Fresh vegetables	1.0	0.9
9	Eggs	2.0	1.8	8	Pork meat	2.3	0.8
10	Breakfast cereals	1.0	1.4	9	Plain yogurts and fermented milk	0.6	0.7
11	Poultry meat	2.6	1.2	10	Bluefish	0.8	0.5
12	Fresh shellfish and mollusks	0.5	1.2	Processed culinary ingredients			
13	Bovine meat	2.4	1.0				
14	Sausages	0.2	1.0	1	Butter	0.7	0.1
15	Fish preserves	0.4	0.9	2	Oils	10.3	0.0
16	Fresh vegetables	1.0	0.9	2	Sugars	0.5	0.0
17	Whitefish	0.6	0.9	3	Lard	0.3	0.0
18	Toasted bread	0.3	0.9				
19	Milkshakes	0.9	0.7	Processed foods and beverages			
20	Hamburger bread	0.4	0.6	1	White bread	6.9	11.6
Discretionary foods and beverages				2	Serrano ham	0.6	8.2
1	Serrano ham	0.6	8.2	3	Cured cold meats	1.8	6.6
2	Cured cold meats	1.8	6.6	4	Semicured and cured cheeses	1.5	2.6
3	Cube soup	0.1	6.1	5	Sugared and with fruit yogurts	2.4	1.4
4	Cold meat	1.6	5.4	6	Canned fish	0.4	0.9
5	Sausages	1.1	3.5	7	Bakery products	0.7	0.6
6	Buns, sweet bread, etc.	5.7	3.4	8	Pickled vegetables	0.1	0.6
7	Biscuits	5.8	2.6	9	Spread cheese and cheese in portions	0.3	0.4
8	Chocolates	3.6	2.1	10	Smoked pork meat	0.4	0.4
9	Snacks	1.5	1.4	11	Fresh cheese	0.2	0.4
10	Sauces	0.7	1.4	Ultra-processed foods and beverages			
11	Breakfast cereals	1.0	0.8	1	Cube soup	0.1	6.1
12	Pre-cooked and ready-to-eat meals	0.5	0.7	2	Cold meat	1.6	5.4
13	Bakery products	0.8	0.7	3	Pizzas	2.1	4.9
14	Pickling vegetables	0.1	0.6	4	Sausages	1.3	4.5
15	Pâté	0.3	0.6	5	Sliced bread	1.9	3.6
16	Semi-fat pork meat and bacon	0.9	0.4	6	Buns, sweet bread, etc.	5.7	3.4
17	Smoked pork meat	0.4	0.4	7	Pre-cooked and ready-to-eat meals	1.6	3.3
18	Surimi	0.1	0.4	8	Biscuits	5.8	2.6
19	Dairy desserts	0.8	0.4	9	Breakfast cereals	1.9	2.1
20	Isotonic drinks	0.2	0.2	10	Chocolates	3.6	2.1

## References

[1] World Health Organization (2012). Guideline: Sodium Intake for Adults and Children.

[2] Dietary Guidelines Advisory Committee (2010). Report of the Dietary Guidelines Advisory Committee on the Dietary Guidelines for Americans, 2010.

[3] Wong M.M.Y., Arcand J.A., Leung A.A., Raj T.S., Trieu K., Santos J.A., Campbell N.R.C. (2017). The Science of Salt: A Regularly Updated Systematic Review of Salt and Health Outcomes (August to November 2015). J. Clin. Hypertens..

[4] World Health Organization (2013). Mapping Salt Reduction Initiatives in the WHO European Region.

[5] EFSA NDA Panel (EFSA Panel on Dietetic Products, Nutrition and Allergies) (2005). Opinion of the Scientific Panel on Dietetic products, nutrition and allergies related to the Tolerable Upper Intake Level of Sodium. EFSA J..

[6] World Health Organization (2003). Diet, Nutrition and the Prevention of Chronic Diseases. Report of a Joint WHO/FAO Expert Consultation.

[7] European Commission (2012). Implementation of the EU Salt Reduction Framework—Results of Member States Survey.

[8] Campanozzi A., Avallone S., Barbato A., Iacone R., Russo O., De Filippo G., D’Angelo G., Pensabene L., Malamisura B., Cecere G. (2015). High sodium and low potassium intake among Italian children: Relationship with age, body mass and blood pressure. PLoS ONE.

[9] AFSSA (Agence française de sécurité sanitaire des aliments) (2017). Étude individuelle nationale des consommations alimentaires 3 (INCA 3).

[10] Agencia Española de Consumo, Seguridad Alimentaria y Nutrición (2014). Informe del Comité Científico de la Agencia Española de Consumo, Seguridad Alimentaria y Nutrición Sobre Objetivos y Recomendaciones Nutricionales y de Actividad Física Frente a la Obesidad en el Marco de la Estrategia NAOS.

[11] Aranceta J., Miján de la Torre A., Moreno Villares J.M. (2005). Clínicas Españolas De Nutrición.

[12] Liem D.G. (2017). Infants’ and Children’s Salt Taste Perception and Liking: A Review. Nutrients.

[13] Mennella J.A., Finkbeiner S., Lipchock S.V., Hwang L.-D., Reed D.R. (2014). Preferences for salty and sweet tastes are elevated and related to each other during childhood. PLoS ONE.

[14] Dossier Resumen: Plan de Colaboración Para de los Alimentos y Bebidas y Otras Medidas 2017–2020. http://www.aecosan.msssi.gob.es/AECOSAN/docs/documentos/nutricion/DOSSIER_PLAN_2020.pdf.

[15] Ahmed A., Ahmad A., Khalid N., David A., Sandhu M.A., Randhawa M.A., Suleria H.A.R. (2014). A Question Mark on Iron Deficiency in 185 Million People of Pakistan: Its Outcomes and Prevention. Crit. Rev. Food Sci. Nutr..

[16] Sultan S., Anjum F.M., Butt M.S., Huma N., Suleria H.A.R. (2014). Concept of double salt fortification; a tool to curtail micronutrient deficiencies and improve human health status. J. Sci. Food Agric..

[17] Grieger J.A., Wycherley T.P., Johnson B.J., Golley R.K. (2016). Discrete strategies to reduce intake of discretionary food choices: A scoping review. Int. J. Behav. Nutr. Phys. Act..

[18] Aparicio A., Rodríguez-Rodríguez E., Cuadrado-Soto E., Navia B., López-Sobaler A.M., Ortega R.M. (2015). Estimation of salt intake assessed by urinary excretion of sodium over 24 h in Spanish subjects aged 7–11 years. Eur. J. Nutr..

[19] (2018). DIAL (for Windows, version 3.0.0.12); DIAL software for assessing diets and food calculations.

[20] Ortega R.M., López-Sobaler A.M., Requejo A.M., Andrés P. (2010). Food Composition. A Basic Tool for Assessing Nutritional Status.

[21] Krebs-Smith S.M., Kott P.S., Guenther P.M. (1989). Mean proportion and population proportion: Two answers to the same question?. J. Am. Diet. Assoc..

[22] O’Halloran S.A., Grimes C.A., Lacy K.E., Nowson C.A., Campbell K.J. (2016). Dietary sources and sodium intake in a sample of Australian preschool children. BMJ Open.

[23] Ziauddeen N., Almiron-Roig E., Penney T., Nicholson S., Kirk S., Page P. (2017). Eating at Food Outlets and “On the Go” Is Associated with Less Healthy Food Choices in Adults: Cross-Sectional Data from the UK National Diet and Nutrition Survey Rolling Programme (2008–2014). Nutrients.

[24] Johnson L., van Jaarsveld C.H., Wardle J. (2011). Individual and family environment correlates differ for consumption of core and non-core foods in children. Br. J. Nutr..

[25] Fayet-Moore F., Petocz P., McConnell A., Tuck K., Mansour M. (2017). The cross-sectional association between consumption of the recommended five food group “Grain (Cereal)”, dietary fibre and anthropometric measures among australian adults. Nutrients.

[26] National Health and Medical Research Council (2013). Australian Dietary Guidelines.

[27] Australian Bureau of Statistics Discretionary Food List—Australian Health Survey: Users’ Guide, 2011–2013. http://www.abs.gov.au/AUSSTATS/abs@.nsf/DetailsPage/4363.0.55.0012011-13?OpenDocument.

[28] Moubarac J.-C. (2015). Ultra-Processed Food and Drink Products in Latin America: Trends, Impact on Obesity, Policy Implications.

[29] Moubarac J.-C., Batal M., Louzada M.L., Martinez Steele E., Monteiro C.A. (2017). Consumption of ultra-processed foods predicts diet quality in Canada. Appetite.

[30] Schofield W.N. (1985). Predicting basal metabolic rate, new standards and review of previous work. Hum. Nutr. Clin. Nutr..

[31] Goldberg G., Black A., Jebb S., Colte T., Murgatroyd P., Coward W., Prentice A. (1991). Critical evaluation of energy intake data using fundamental principles of energy physiology: 1. Derivation of cut-off limits to identify under-recording. Eur. J. Clin. Nutr..

[32] Moosavian S.P., Haghighatdoost F., Surkan P.J., Azadbakht L. (2017). Salt and obesity: A systematic review and meta-analysis of observational studies. Int. J. Food Sci. Nutr..

[33] Black A. (2000). Critical evaluation of energy intake using the Goldberg cut-off for energy intake:basal metabolic rate. A practical guide to its calculation, use and limitations. Int. J. Obes..

[34] Colin-Ramirez E., Espinosa-Cuevas Á., Miranda-Alatriste P.V., Tovar-Villegas V.I., Arcand J., Correa-Rotter R. (2017). Food Sources of Sodium Intake in an Adult Mexican Population: A Sub-Analysis of the SALMEX Study. Nutrients.

[35] López-Sobaler A.M., Aparicio A.A., González-Rodríguez L.G., Cuadrado-Soto E., Rubio J., Marcos V., Sanchidrián R., Santos S., Pérez-Farinós N., Ángeles M. (2017). Adequacy of Usual Vitamin and Mineral Intake in Spanish Children and Adolescents: ENALIA Study. Nutrients.

[36] Hu F.B. (2008). Obesity Epidemiology.

[37] Vainik U., Konstabel K., Lätt E., Mäestu J., Purge P., Jürimäe J. (2016). Diet misreporting can be corrected: Confirmation of the association between energy intake and fat-free mass in adolescents. Br. J. Nutr..

[38] Desor J.A., Greene L.S., Maller O. (1975). Preferences for sweet and salty in 9- to 15-year-old and adult humans. Science.

[39] Jaenke R., Barzi F., McMahon E., Webster J., Brimblecombe J. (2017). Consumer acceptance of reformulated food products: A systematic review and meta-analysis of salt-reduced foods. Crit. Rev. Food Sci. Nutr..

[40] Marrero N.M., He F.J., Whincup P., MacGregor G.A. (2014). Salt intake of children and adolescents in south London consumption levels and dietary sources. Hypertension.

[41] Eloranta A.M., Venäläinen T., Soininen S., Jalkanen H., Kiiskinen S., Schwab U., Lakka T.A., Lindi V. (2016). Food sources of energy and nutrients in Finnish girls and boys 6–8 years of age—The PANIC study. Food Nutr. Res..

[42] Gaitán D.A., Estrada A., Lozano G.A., Luz Y., Manjarres M. (2015). Food sources of sodium: Analysis Based on a national survey in Colombia. Nutr. Hosp..

[43] Grimes C.A., Campbell K.J., Riddell L.J., Nowson C.A. (2011). Sources of sodium in Australian children’s diets and the effect of the application of sodium targets to food products to reduce sodium intake. Br. J. Nutr..

[44] Fayet-Moore F., Pearson S. (2015). Interpreting the Australian dietary guideline to “limit” into practical and personalised advice. Nutrients.

[45] Sarmugam R., Worsley A. (2014). Current Levels of Salt Knowledge: A Review of the Literature. Nutrients.

[46] Aranceta Bartrina J., Arija Val V., Maíz Aldalur E., Martínez de Victoria Muñoz E., Ortega Anta R.M., Pérez-Rodrigo C., Quiles Izquierdo J., Rodríguez Martín A., Román Viñas B., Salvador Castell G. (2016). Guías alimentarias para la población española (SENC, diciembre 2016); la nueva pirámide de la alimentación saludable. Nutr. Hosp..

[47] Magriplis E., Farajian P., Pounis G.D., Risvas G., Panagiotakos D.B., Zampelas A. (2011). High sodium intake of children through “hidden” food sources and its association with the Mediterranean diet: The GRECO study. J. Hypertens..

[48] Gonçalves C., Abreu S., Padrão P., Pinho O., Graça P., Breda J., Santos R., Moreira P. (2016). Sodium and potassium urinary excretion and dietary intake: A cross-sectional analysis in adolescents. Food Nutr. Res..

[49] Regional Expert Group for Cardiovascular Disease Prevention through Population-Wide Dietary Salt Reduction 2010. https://www.paho.org/hq/dmdocuments/2013/Metodos-determinar-fuentes-sodio-Eng.pdf.

[50] Dötsch-Klerk M., Goossens W.P., Meijer G., Van Het Hof K. (2015). Reducing salt in food; setting product-specific criteria aiming at a salt intake of 5 g per day. Eur. J. Clin. Nutr..

